# Integrative analysis of microbiota and metabolomics in chromium-exposed silkworm (*Bombyx mori*) midguts based on 16S rDNA sequencing and LC/MS metabolomics

**DOI:** 10.3389/fmicb.2023.1278271

**Published:** 2023-10-25

**Authors:** Ya-Zhen Chen, Wan-Tao Rong, Ying-Can Qin, Lin-Yuan Lu, Jing Liu, Ming-Jie Li, Lei Xin, Xiao-Dong Li, De-Long Guan

**Affiliations:** ^1^Guangxi Key Laboratory of Sericulture Ecology and Applied Intelligent Technology, Hechi University, Hechi, China; ^2^Guangxi Collaborative Innovation Center of Modern Sericulture and Silk, Hechi University, Hechi, China

**Keywords:** chromium exposure, silkworms, gut microbiota, metabolomics, multi-omics

## Abstract

The gut microbiota, a complex ecosystem integral to host wellbeing, is modulated by environmental triggers, including exposure to heavy metals such as chromium. This study aims to comprehensively explore chromium-induced gut microbiota and metabolomic shifts in the quintessential lepidopteran model organism, the silkworm (*Bombyx mori*). The research deployed 16S rDNA sequence analysis and LC/MS metabolomics in its experimental design, encompassing a control group alongside low (12 g/kg) and high (24 g/kg) feeding chromium dosing regimens. Considerable heterogeneity in microbial diversity resulted between groups. *Weissella* emerged as potentially resilient to chromium stress, while elevated Propionibacterium was noted in the high chromium treatment group. Differential analysis tools LEfSe and random forest estimation identified key species like like *Cupriavidus* and *unspecified Myxococcales*, offering potential avenues for bioremediation. An examination of gut functionality revealed alterations in the KEGG pathways correlated with biosynthesis and degradation, suggesting an adaptive metabolic response to chromium-mediated stress. Further results indicated consequential fallout in the context of metabolomic alterations. These included an uptick in histidine and dihydropyrimidine levels under moderate-dose exposure and a surge of gentisic acid with high-dose chromium exposure. These are critical players in diverse biological processes ranging from energy metabolism and stress response to immune regulation and antioxidative mechanisms. Correlative analyses between bacterial abundance and metabolites mapped noteworthy relationships between marker bacterial species, such as *Weissella* and *Pelomonas*, and specific metabolites, emphasizing their roles in enzyme regulation, synaptic processes, and lipid metabolism. Probiotic bacteria showed robust correlations with metabolites implicated in stress response, lipid metabolism, and antioxidant processes. Our study reaffirms the intricate ties between gut microbiota and metabolite profiles and decodes some systemic adaptations under heavy-metal stress. It provides valuable insights into ecological and toxicological aspects of chromium exposure that can potentially influence silkworm resilience.

## Introduction

The silkworm (*Bombyx mori*), treasured for its capacity to produce versatile silk, upholds an integral place in various sectors, notably the textile industry (Huang W. et al., 2018; Bucciarelli and Motta, 2022). Places like Guangxi, China, see their local economy deeply intertwined with this economically critical insect, reflecting the living standards of farmers and workers involved with silk (Liang et al., 2013; Chen et al., 2022; Zhao et al., 2022). Intending to enhance farming efficacy, economic value, and quality of life for these communities, diligent scientific research and productive rearing practices constitute an indispensable step. However, heavy metal toxicity is a perilous interruption to silkworm farming, particularly in industry-dense regions such as Guangxi (Li et al., 2021; Xu et al., 2022). Heavy metals such as cadmium, chromium, and Arsenic harm the silkworm’s average growth and silk production lifeline, often leading to death and indenting substantial economic loss (Du et al., 2021; Li et al., 2021; Zang et al., 2023). Recent scientific reviews culminated in supporting these concerns, outlining that heavy metal toxicity disturbs essential biological processes pivotal for the silkworm’s lifecycle and silk production (Wan et al., 2017; Marzoli et al., 2022; Gao et al., 2023).

Chromium, a heavy metal ubiquitous in the environment, poses remarkable threats to the environment and life systems due to its widespread use in various industrial activities. Previous works on chromium’s biological toxicity extend the spectrum of affected organisms, encompassing humans, animals, model organisms, and insects (Mishra and Bharagava, 2016; Rahman and Singh, 2019; Kapoor et al., 2022). Established research highlights adverse effects such as DNA damage, oxidative stress, and the induction of apoptosis (Balali-Mood et al., 2021; Chakraborty et al., 2022; Teschke, 2022). Mainly, apoptosis, a programmed cellular death, occurs consequent to a cascade of intracellular events activated under stressful conditions (Chiu et al., 2010; Balali-Mood et al., 2021; Renu et al., 2021). This potent mechanism, generally a protective action against teratogenesis, under excessive exposure can bring about damage, disease, or even organismal death.

Focusing on the silkworm milieu, chromium toxicity brings with it a profound hindrance to average growth, development, and silk production. However, this forms only part of our understanding, and the breadth of its results remains under-researched. The implications of chromium in non-tissue microenvironments such as the gut or silk glands have been less frequently analyzed (Muhammad et al., 2022; Chen et al., 2023). The silkworm gut microbiota, an indispensable entity within the silkworm physiology, plays a fundamental role in contributing to the worm’s health and life activity. In the face of chromium toxicity, these microbial communities are expected to undergo dynamic shifts, potentially embodying a secondary environmental stressor for silkworms. This biotic focus on gut ecology assumes great relevance, considering its critical role in disease prevention, digestion, nutrient absorption, and apt synthesis of silk (Li et al., 2021; Li C. et al., 2022; Xu et al., 2022).

While growing scientific capabilities, novel methodologies have emerged at the intersection of microbiology and genomics; incorporating metagenomic sequencing and non-targeted metabolomics is one such leap (Dong et al., 2019; Debnath et al., 2021; Jia et al., 2021; Coker et al., 2022). In our investigation, we deploy robust 16S rDNA microbial metagenomic sequencing to gain a comprehensive image of gut microbiota composition. We couple this with LC/MS non-targeted metabolomics, aimed at widening the understanding of functionally significant metabolites in the gut. Together, these cutting-edge techniques promise powerful, high-resolution insights, allowing us to traverse both biodiversity and functional aspects within the microbiome.

Our outlined course of inquiry, exploring the impacts of chromium on the intricate gut ecology of silkworms at a diet exposure concentration of 12 g/kg and 24 g/kg, poses a monumental stride in grasping the broad scope of chromium toxicity. This investigation brings promise in elaborately resolving how chromium shapes the gut microbiota and metabolome, bridging existing knowledge gaps and directing future focus. Most meaningfully, this study could potentially reform the understanding of heavy metal toxicity, underpin a reasoned approach toward heavy metal pollution mitigation and propel sustainable practices in silkworm farming. With its far-reaching implications, we hope to inspire further research endeavors, setting the stage for application-driven initiatives to bolster the health and productivity of economically significant organisms, like silkworms, in at-risk ecological niches, thereby supporting the resilience and livelihood of human communities largely dependent on them.

## Materials and methods

### Sample collection

The silkworm samples utilized in this research were collected from the Gui Can No.5 string. This strain has been under long-term cultivation in the laboratory of Hechi University. Three groups were established for this experiment. Each group consists of six samples, each comprising at least 10 individuals. The control group (ACB) was fed a regular non-chromium-added artificial diet. Two experimental groups consisted of the middle group and the high group, which received feed containing Chromium chloride at concentrations of 12 g/kg (group B2B, 50% half-lethal dose at 5 days) and 24 g/kg (group C4B, half-lethal dose at 5 days), respectively. After 120 h (5 days) at stage five larval stage, entire midgut tissues were collected from 10 randomly selected live individuals from each experimental group. The tissue samples were dissected on ice and then stored at −80°C in preparation for subsequent 16s rDNA extraction and sequencing analysis.

### 16S rDNA gene sequencing

The total genomic DNA was extracted employing the hexadecyl trimethyl ammonium bromide (CTAB)/sodium dodecyl sulphate (SDS) method (Ramimoghadam et al., 2012; Xia et al., 2019). DNA quantification and purity were monitored using 1% agarose gel electrophoresis. The DNA samples were diluted to 1 ng/μl using sterile distilled water. Amplification of the 16S rDNA gene was carried out with primers specific for 515F – 806R (V3-V4) regions (Parada et al., 2016; Wurm et al., 2023). The PCR products were quantified and visualized by performing 2% agarose gel electrophoresis with 1× loading buffer containing SYBR Green. A sequencing library was prepared using the NEB Next Ultra DNA Library Prep Kit (Illumina; NEB, USA). Index codes were added, and quality checks were completed using a Qubit 2.0 Fluorometer (Thermo Scientific, USA) and an Agilent BioAnalyzer 2,100 system (Agilent Technologies, Palo Alto, CA). Finally, the Illumina MiSeq platform (Illumina, NEB, USA) was used for sequencing, producing 300 bp paired-end reads.

### 16S rDNA statistical analysis

Paired raw reads of all 18 samples were filtered using Trimmomatic v0.38 (Bolger et al., 2014) to remove low-quality reads, including those containing adapters and contaminants. Using a custom-made script, We further excluded reads with a Phred quality score below 20 (Q20 < 90) and a total length of fewer than 50 base pairs. With the high-quality reads secured, we conducted further statistical analysis in the software environment of QIIME II (version 2.01) (Hall and Beiko, 2018; Fung et al., 2021). The DADA2 method was applied for primer trimming, quality filtering, denoising, merging, and chimera removal in the study (Callahan et al., 2016). Initially, the QIIME cut-adapt trim-paired tool removed the primer sequences and discarded the unmatched sequences. Subsequently, DADA2 was employed using the QIIME DADA2 denoise-paired command to perform quality control, denoising, merging, and chimera removal. These steps were performed separately for each library. After denoising all libraries, the ASVs’ (amplicon sequence variants) feature sequences and ASV table were merged, and singleton ASVs (ASVs with only one sequence in the entire set of samples) were removed by default. The distribution of sequence lengths was calculated using an R script, considering the high-quality sequences in all samples. The ASVs generated after DADA2 quality control are currently highly recommended by QIIME2. ACB, B2B and C4B group names were assigned to the control, moderate concentrate treatment group of 12 g/kg, and high concentrate group of 24 g/kg, respectively. Each group comprise six independent samples.

We employed various ecological diversity indices to thoroughly examine the alpha diversity of the microbial communities in our samples (Hall and Beiko, 2018; Fung et al., 2021). To determine community richness, the Chao1 and Observed Species indices were used. The Shannon and Simpson indices were employed in quantifying community diversity. We used Faith’s Phylogenetic Diversity (PD) index to express evolutionary diversity. Pielou’s evenness index was calculated to estimate community evenness, and Good’s coverage index was used to assess the coverage of our survey. The statistics for alpha diversity indices are provided in Supplementary File S1.

Downstream analyses included principal component analysis, random forest estimation, UPGMA clustering, and Venn diagram generation, performed using the PersonalBio online platform.^1^ PCoA (Principal Coordinate Analysis) analysis was performed using the rarefied ASV table. The “qiime diversity core-metrics-phylogenetic” command was used to calculate four distance matrices: Jaccard, Bray-Curtis, unweighted UniFrac, and weighted UniFrac. These distance matrices were then transformed into sample distances and projected onto a two-dimensional plot. The PCoA results were evaluated based on the explained variance, and the top two components (PCoA1 and PCoA2) with the highest proportion of variation were selected. This study chose the weighted UniFrac distance matrix as it explained the highest proportion of variation. Functional capabilities of the microbiota were evaluated using the PICRUSt2 software (Douglas et al., 2020) to predict the Kyoto Encyclopedia of Genes and Genomes (KEGG) metabolic pathway annotations. Statistical significance (*p*-values) and false discovery rates (FDRs) were calculated using R-based scripts. The statistical tables of microbial taxa abundance at the genus level, principal component analysis sample scores, random forest analysis feature importance, taxonomical KEGG secondary functional statistics, and different KEGG pathways were shown in Supplementary Files S2–S6.

### Non-targeted metabolomics sample preparation and analysis

The same experimental design was adopted for the non-targeted metabolomics, where 18 gut samples under the same chromium treatment were collected and named accordingly using ACB, B2B and C4B. A total of 100 μL of thawed tissue samples were treated with 400 μL of precooled methanol-acetonitrile solution (1:1, v/v), vortexed for 60 s, precipitated for one h at −20°C, and centrifuged at 14,000 g and 4°C for 20 min. Then, the supernatant was freeze-dried before storage at −80°C for further testing (Johnson et al., 2016; Schrimpe-Rutledge et al., 2016; Bauermeister et al., 2022).

### UHPLC-QTOF/MS statistical analysis

Utilizing ProteoWizard (version 3.0.4146) (Kessner et al., 2008; Holman et al., 2014), original data was converted into an mzXML format, following which the XCMS software was adopted for peak alignment, retention time correction, and peak area extraction (Mahieu et al., 2016; Domingo-Almenara and Siuzdak, 2020). Multivariate analyses such as Pareto-scaled PCA and orthogonal partial least squares discriminant analysis (OPLS-DA) were carried out using the software SIMCA – P (version 14.1) (Wheelock and Wheelock, 2013; Wu et al., 2019). Metabolites with variable importance in projection (VIP) value greater than one were subjected to a two-sample Student’s t-test to determine their significance. The statistical tables of metabolites number at the superclass level, the qualitative collation summary for positive and negative ions, differential metabolites, and enriched KEGG pathways for differential metabolites were shown in Supplementary Files S7–S10.

### Omics association analysis

The principal component analysis (PCA) and Co-Inertia Analysis (CIA) were employed to validate the congruence between microbiome and metabolome profiles. Differentially abundant metabolites were subjected to correlation analysis using Spearman correlation. The associations between bacterial taxa and metabolites were identified using 16S rDNA sequencing data. The R language (Kellogg et al., 2020) and Mothur (Chappidi et al., 2019) software were combined to analyze previously mentioned PCA and CIA analyses, draw the matrix heat map, hierarchical clustering, association network, and related parameters. The final correlation between the final differential microbiota and metabolites was obtained through multiple rounds of screening and calculations.

## Results

### Alpha and beta – diversity

**Figure 1:**
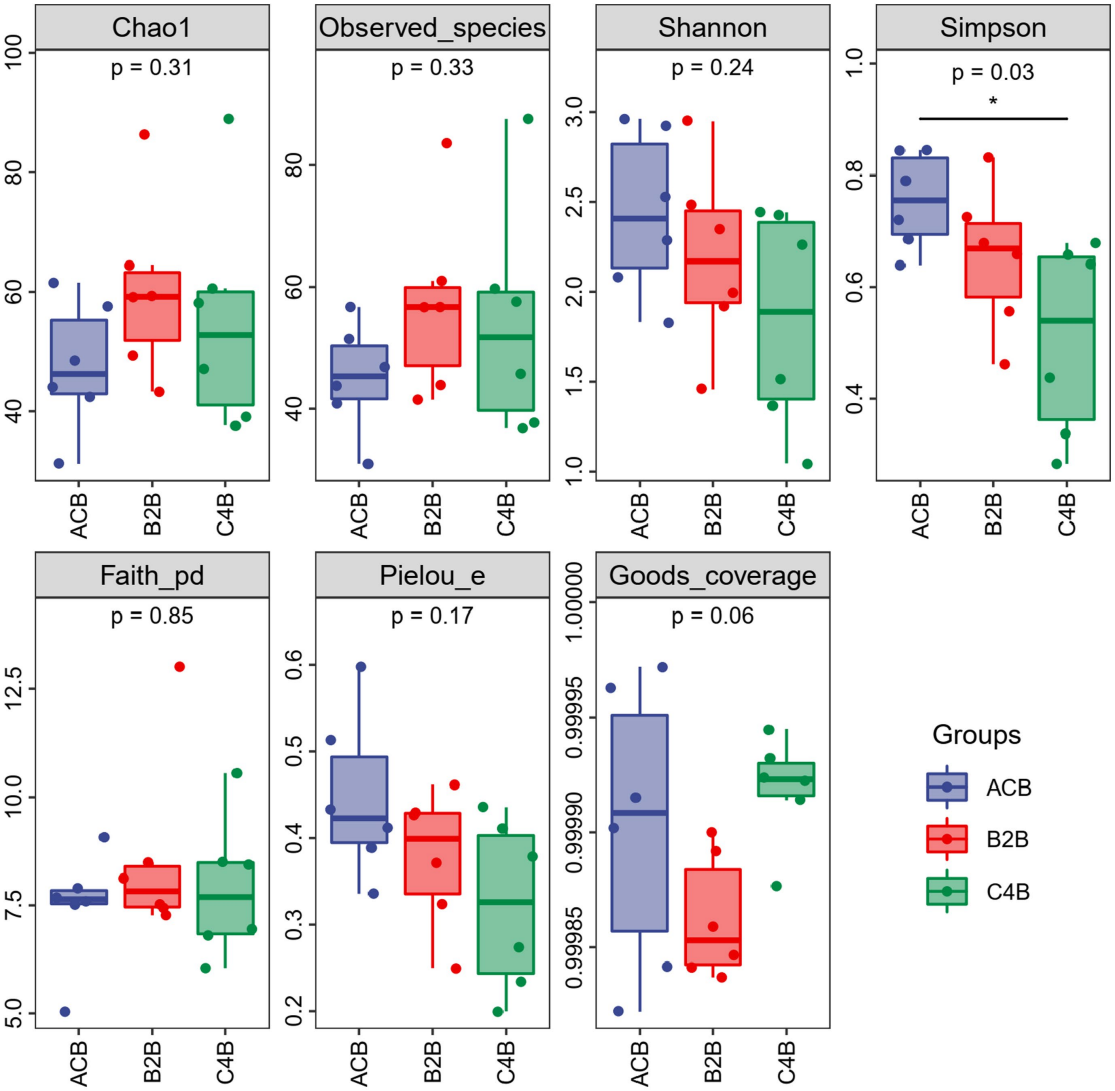
Box plot of alpha diversity indices among different treatment groups.

Through comparison and analysis of alpha diversity parameters across different chromium stress treatments in silkworms’ gut microbiota, we can observe the impact of chromium on the gut microbiota of these organisms (Figure 1). First of all, Goods_coverage, a measure of sequence coverage represents how well the true diversity of each sample, maintained a consistency close to 1 across all groups which mean each group was well determined and characterized. According to the data, the ACB group displayed an overall higher Chao1, Faith_pd, Observed_species, and Shannon, suggesting a more diverse gut microbiota than the chromium-exposed groups (B2B and C4B). With increasing chromium concentration (from B2B to C4B), there was a decline in diversity indices, such as Chao1, Faith_pd, Observed_species, and Shannon index, implying a negative impact of chromium stress on the richness and evenness of gut microbiota (Supplementary File S1). Although the B2B group had a decline in diversity metrics compared to ACB, it showed more pronounced diversity than C4B, pointing to a dose-dependent reduction of microbiota diversity under chromium stress. The Pielou_e and Simpson also revealed decreased evenness and dominance in the gut microbiota due to chromium stress. Thus, it can be inferred that chromium disrupts the gut microbiota composition, potentially affecting the abiotic stress response of silkworms.

**Figure 2:**
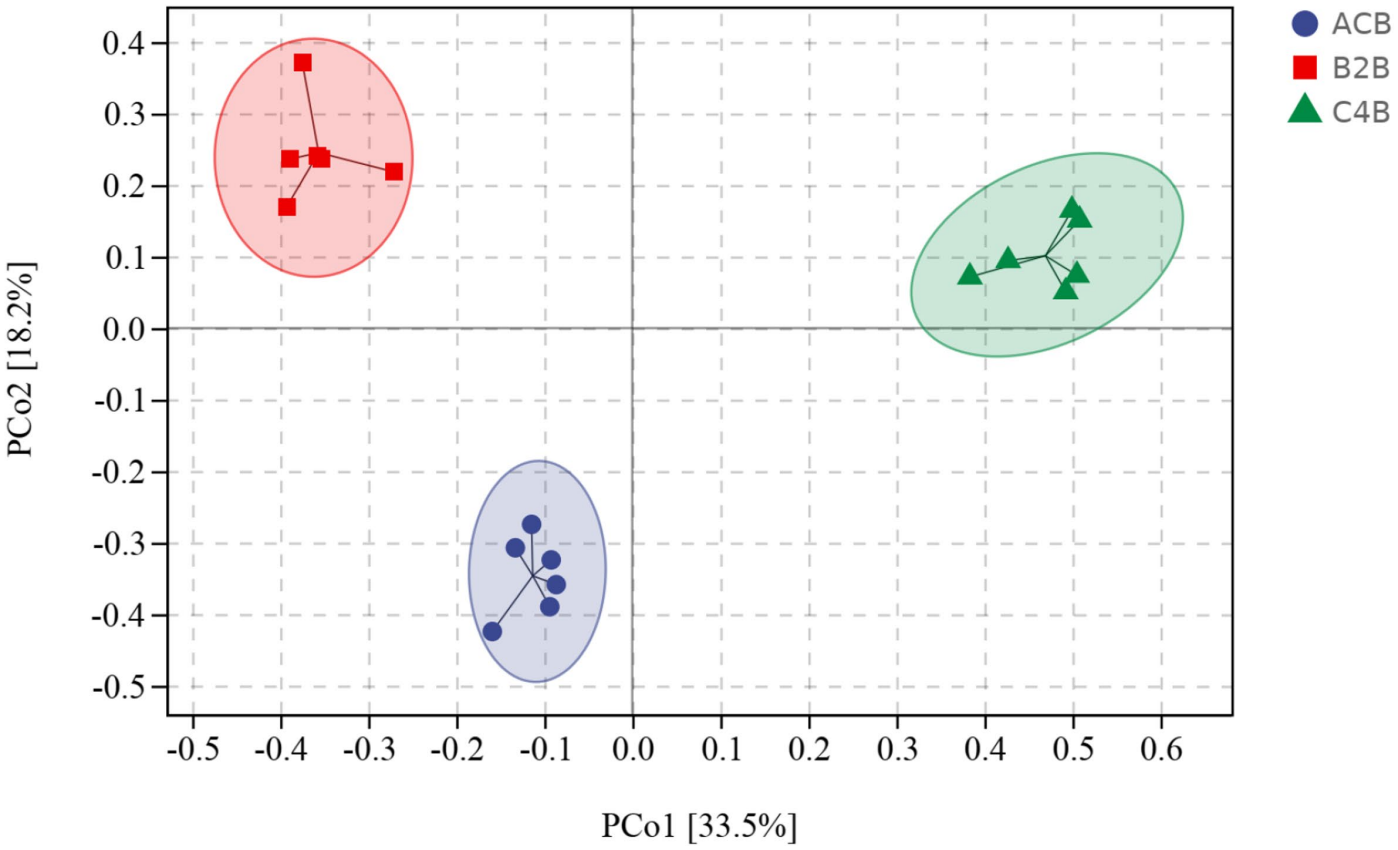
PCoA plot denotes the distance between samples integral. Sample distances were derived from a weighted UniFrac distance matrix.

Beta diversity indexes focus on the differences among samples. Principal coordinates analysis (PCoA), a classical method of non-metric multidimensional scaling analysis that considers the overall distance of samples, is well-suited to the characteristics of ecological data and was thus utilized as the primary analysis tool in this study. The PCoA distance matrix generated from the UniFrac distance matrix explained the highest variation proportion, revealing distinct microbial community profiles among the ACB, B2B, and C4B (Supplementary File S2). Intriguingly, more remarkable dissimilarities were observed between ACB and B2B/C4B, suggesting chromium stress alters silkworms’ intestinal microbiota. Specifically, as the chromium concentration increased, silkworms’ gut microbiota underwent noticeable shifts, as depicted by the pairwise distances (Figure 2). This implies the substantial impact of heavy metal stress on the stability of gut microbiota, potentially leading to the dysbiosis of the bacterial community. The reproducibility of beta diversity within each group (ACB, B2B, C4B) indicates the robustness of biological conclusions derived from these data. Additionally, an increasing trend in PCoA distances in response to chromium concentration suggests a potential dose-dependent impact of chromium on silkworm gut microbiota. Nevertheless, this observation needs further corroboration through statistically rigorous analyses.

**Figure 3:**
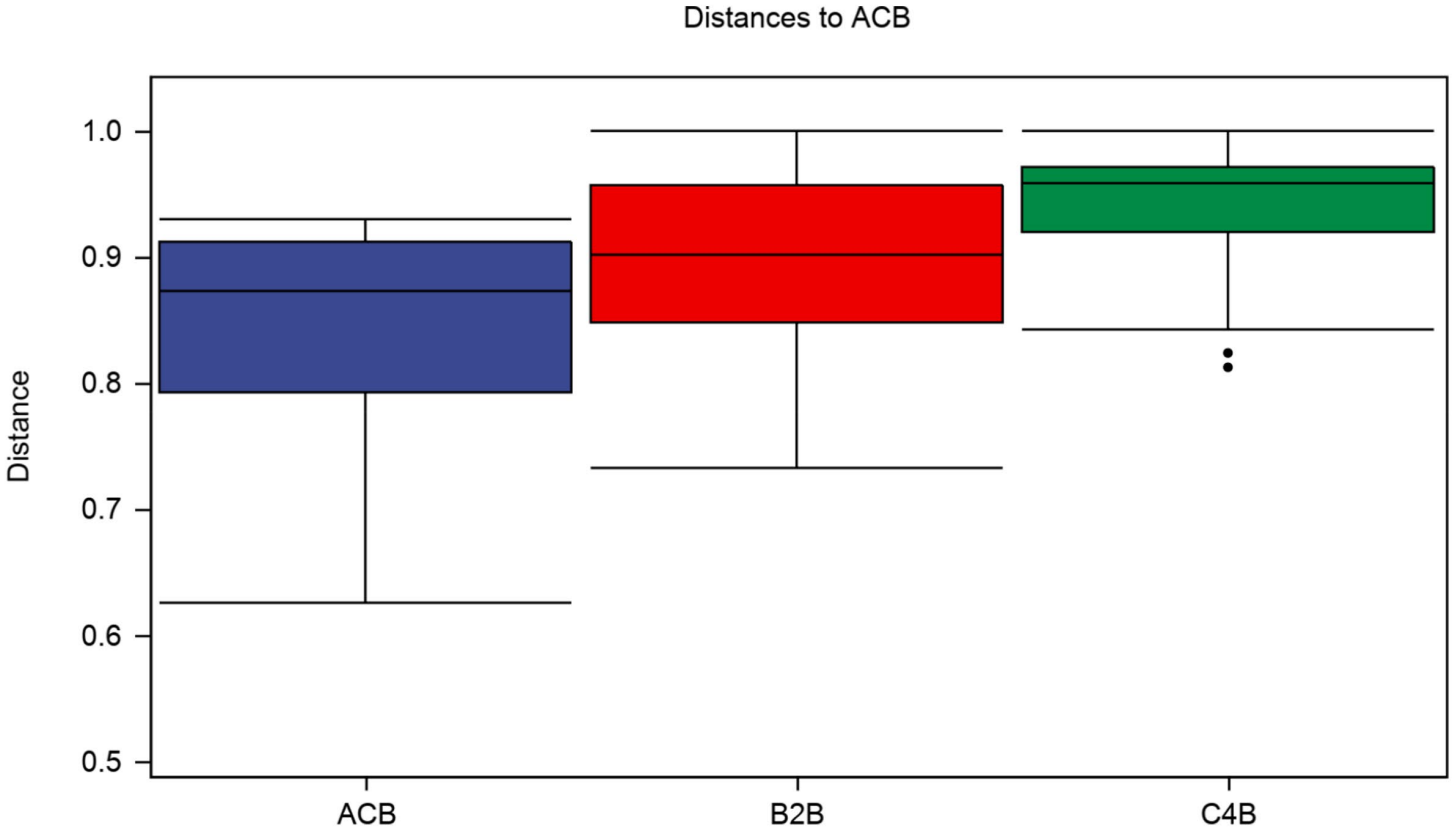
Box plot illustrates the distributions of intra-group and inter-group distances between the sample within each particular group and the samples within the rest of the groups. The top and bottom lines of the box denote the upper and lower quartiles (Interquartile range); the median line signifies the median of the sample groups, the edge lines above and below the box detect the maximum and minimum values; any data points outside these edge-lines are considered as outliers.

In a multidimensional comparison among groups ACB, B2B, and C4B, according to data drawn from permutation tests represented as distances of samples, there are evident differences to note between each. A diversified Interquartile range typically reflects increased differentiation between individual categories (Figure 3). Group ACB provided a baseline indicative of normal intestinal microbiota functioning in silkworms without chromium intervention. In contrast, groups B2B and C4B show evidence of a significant shift in intestinal microbiota under chromium stress, as gleaned from permutation analysis with Q-values (adjusted *p*-values) at 0.003 for ACB vs. B2B, ACB vs. C4B, and B2B vs. C4B. Since the Q-values are less than 0.05, these differences are statistically significant, demonstrating chromium’s substantial impact on the silkworm gut microbiome. Moreover, it is observable that as the chromium pressure increases from B2B to C4B, the interquartile range narrows within each group while the median values elevate culminating at a striking over 0.9 in both the B2B and C4B groups (Figure 3). Implying that a tremendous variation occurs between the moderate and high chromium-stressed groups, it can be deduced that chromium exposure potentially has a dose-responsive influence on silkworm gut microbiota. Such a biological trend, if further affirmed, could have profound implications for understanding chromium intervention effects on other similar biological systems.

### Structural diversity of silkworms’ midgut microbiota

**Figure 4:**
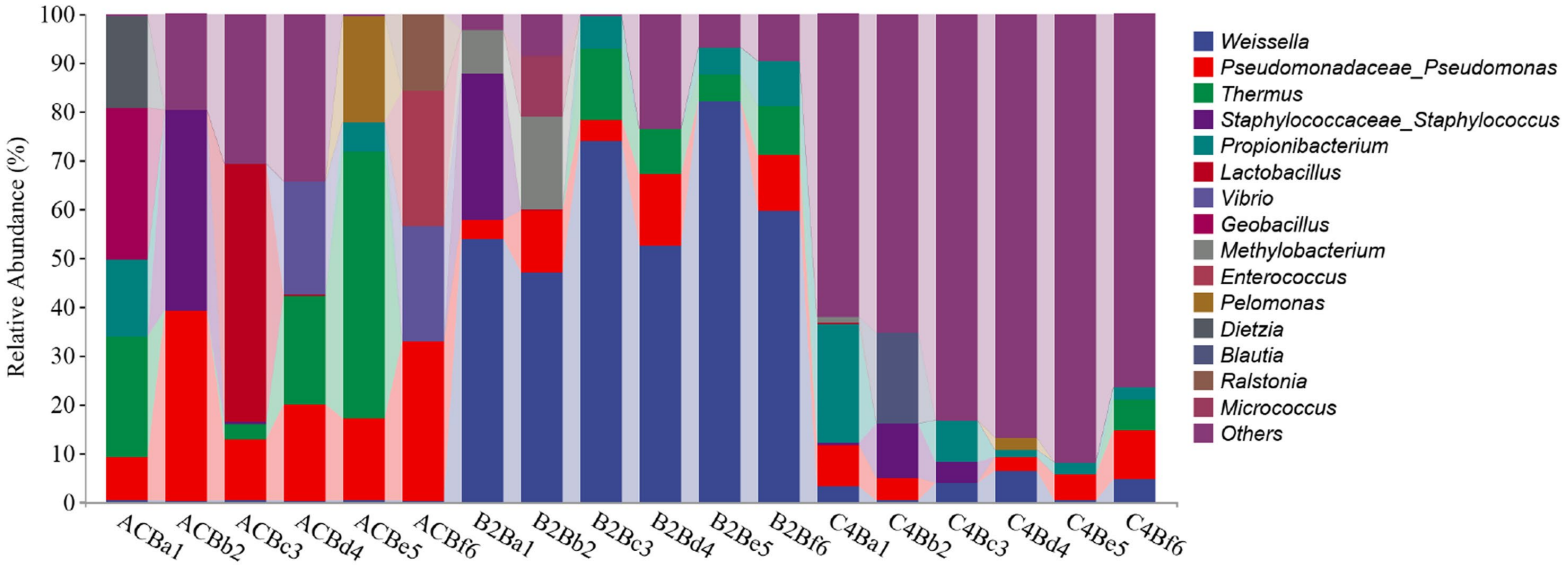
Bar plot presented delineates the taxonomic distribution of bacteria across three different groups treated with varying concentrations of chromium.

Upon treating silkworms with varying concentrations of chromium, we observed variations in the taxonomic distribution of gut bacteria among three different groups (Figure 4). The ACB group, which serves as the control group, displays a diverse microbial community, including *Thermus*, *Geobacillus*, *Dietzia*, and *Propionibacterium* as primary constituents. *Weissella’s* occurrence in this group stays remarkably low. In contrast, the B2B group subjected to medium chromium concentration presents a dominance of *Weissella*, with an evident decline in Thermus and nearly no presence of *Geobacillus* and *Dietzia*, illustrating a prominent shift in the microbial community structure that can be attributed to the chromium stress. The notable company of *Methylobacterium* and *Micrococcus* also marks this group. The C4B group, subjected to high chromium concentration, shows an interesting adaptive microbial response. While a minor percentage still includes *Weissella*, the dominating species becomes unclassified (“Others”) – a factor that suggests a potential emergence of chromium-resistant, uncategorized microorganisms under high mental stress (Figure 4). Propionibacterium exhibits a solid presence alongside elevated levels of *Blautia*. The noted microbiome alterations under varying chromium concentrations could indicate varying degrees of metal tolerance among different microbes, pointing toward adaptive survival strategies under stress. One of the key factors influencing the shift in bacterial diversity may be the variability in biochemical transformations that different strains can activate in the presence of chromium (Supplementary File S3). The predominance of certain species, like *Weissella* in B2B and unclassified organisms in C4B, might be due to their potential chromium-reducing capacities.

### Gut microbiota changes and identification of icon species

This study aimed at understanding the distinct microbial taxa that account for the observed disparities among various groups and clarifying the mechanisms that shape gut microbiota structure. We utilized a comprehensive analytical approach, leveraging supervised and unsupervised learning methodologies. This enabled us to classify microbial communities effectively, pinpoint key taxa responsible for observed variations, and explore the mechanisms structuring gut microbiota across different groups. For example, we employed PCA, an unsupervised learning method, to condense our data and simplify the representation of intricate microbial communities, discerning primary trends and patterns. We also used Random Forests, a supervised learning method, to create predictive models based on microbial composition patterns. RFs adeptly handled high-dimensional, multicollinearity data, thereby identifying essential microbial taxa that essentially explained group disparities. Finally, we used Linear discriminant analysis Effect Size (LEfSe) to combine statistical significance with biological relevance. This approach aided us in identifying microbial taxa that could act as biomarkers for different groups.

**Figure 5:**
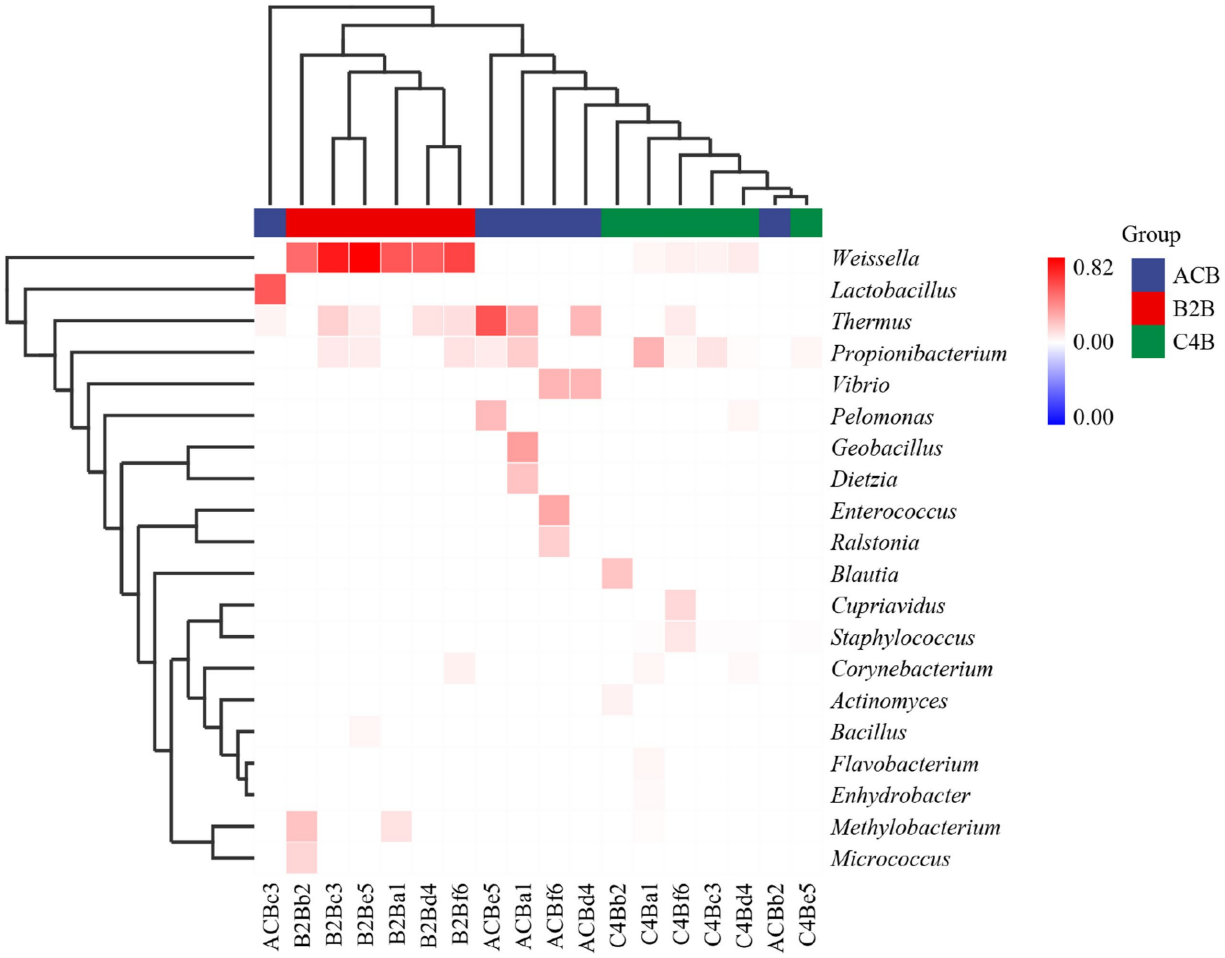
Heatmap cluster map shows the chart of microbiome abundance. The single cluster method was used for both the rows and columns.

The observed heatmap underscores the integral role that chromium plays in molding the bacterial assembly within the gut microbiota inhabiting silkworms (Figure 5). As depicted in Figure 4; the heatmap provides a clear visual representation of the composition and relative abundance of different microbial taxa within each group. The consistency of microbial taxa across multiple samples and the microbial compositions within each group were accessed through cluster analysis of columns and rows. Within the dimensions of the ACB group, the genera *Thermus*, *Geobacillus* and *Bacillus* assert dominance among the battery of taxonomic clusters, insinuating their substantial role in maintaining the unperturbed physiological state of silkworms under conditions devoid of environmental stressors. Concurrently, the onset of moderate chromium stress in the B2B group triggers a remarkable amplification in the population density of *Weissella*, accompanied by a concurrent diminution in the prevalence of *Thermus*. This raises the potential hypothesis of chromium resistance or detoxifying capability vested within the *Weissella* genus. In stark contrast, within the high chromium exposure group C4B, the relative abundance of *Propionibacterium* displays a marked increase, parallel to a simultaneous dwindling of *Weissella* (Figure 5). Such differential microbial responses propound potential adaptive modifications under diverse chromium concentrations. Intriguingly, the emergence of specific icon bacterial groups, such as *Weissella* and *Cupriavidus*, associated with robust heavy metal affinity portends a prospective biochemical remediation tactic employed by the gut microbiota.

**Figure 6:**
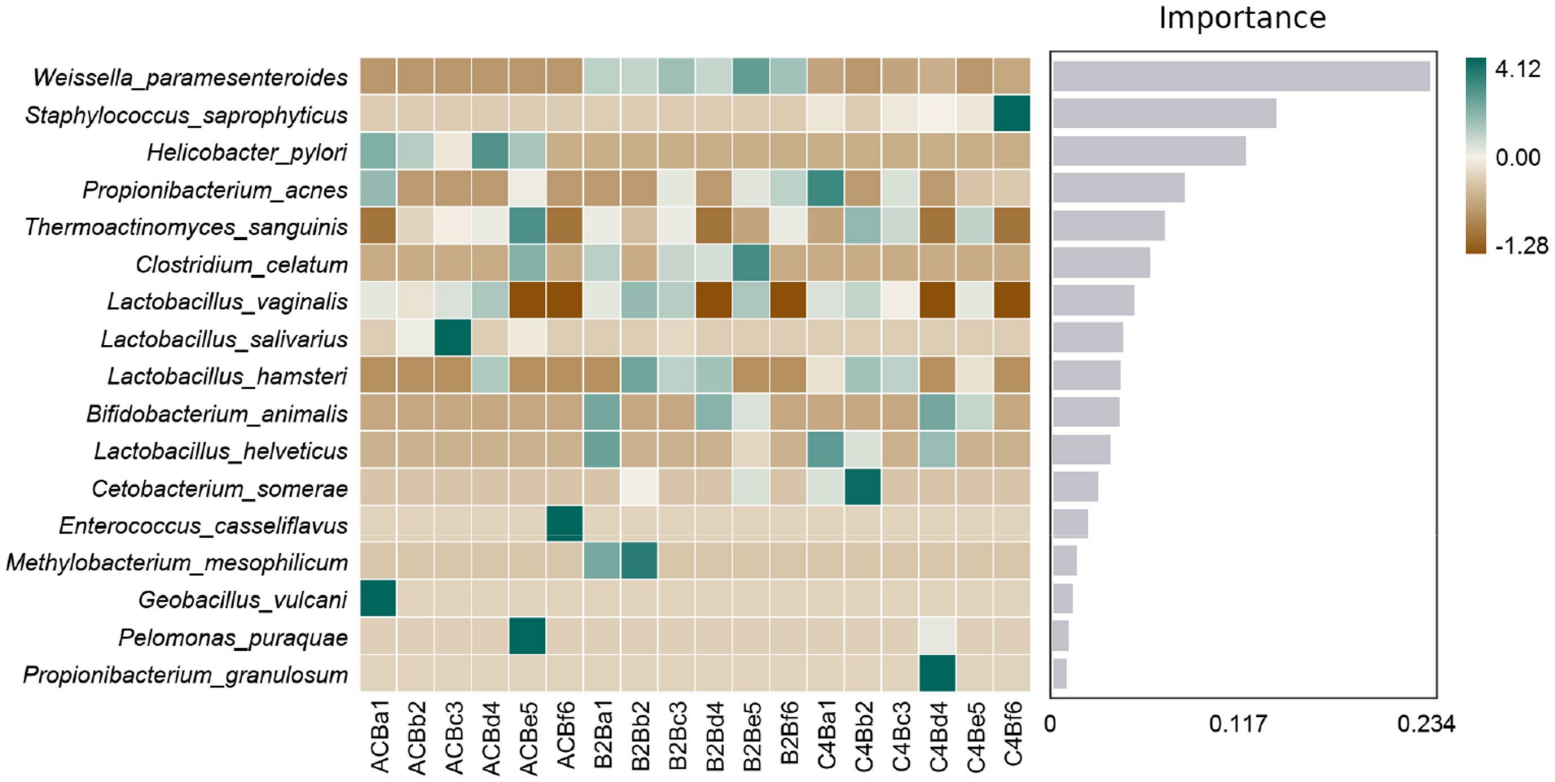
Heatmap illustrating the abundance distribution of species across different samples/groups. Species are ranked from top to bottom based on their importance to the model, with the significance diminishing progressively.

Subsequently, our comparative analysis elucidates the complex interplay between microbial representatives and varying chromium stress conditions, as mapped in the context of Random Forest feature importance (Supplementary File S4). The centerpiece of our findings lies in the significant cardinal shifts in microbial populations across these groups. The markedly heightened prevalence of *Weissella paramesenteroides* in both chromium-stressed groups, B2B and C4B, when contrasted with the control group, underpins a plausible endurance or functional adaptation in the face of chromium stress (Figure 6). Contrarily, *Enterococcus casseliflavus*, conspicuous in its presence within the control environment, is wholly absent in chromium-stressed groups — hinting at a possible vulnerability to chromium stress. As we delve deeper, an intriguing narrative unfolds around *Propionibacterium acnes*; its numbers surge within the C4B group while maintaining constancy in the B2B group (Figure 6). Catching one’s attention is the high prevalence of this bacteria under control conditions, which, unfortunately, meets with a dramatic decline in B2B; the trend, however, sees a relative recovery within the C4B group.

**Figure 7:**
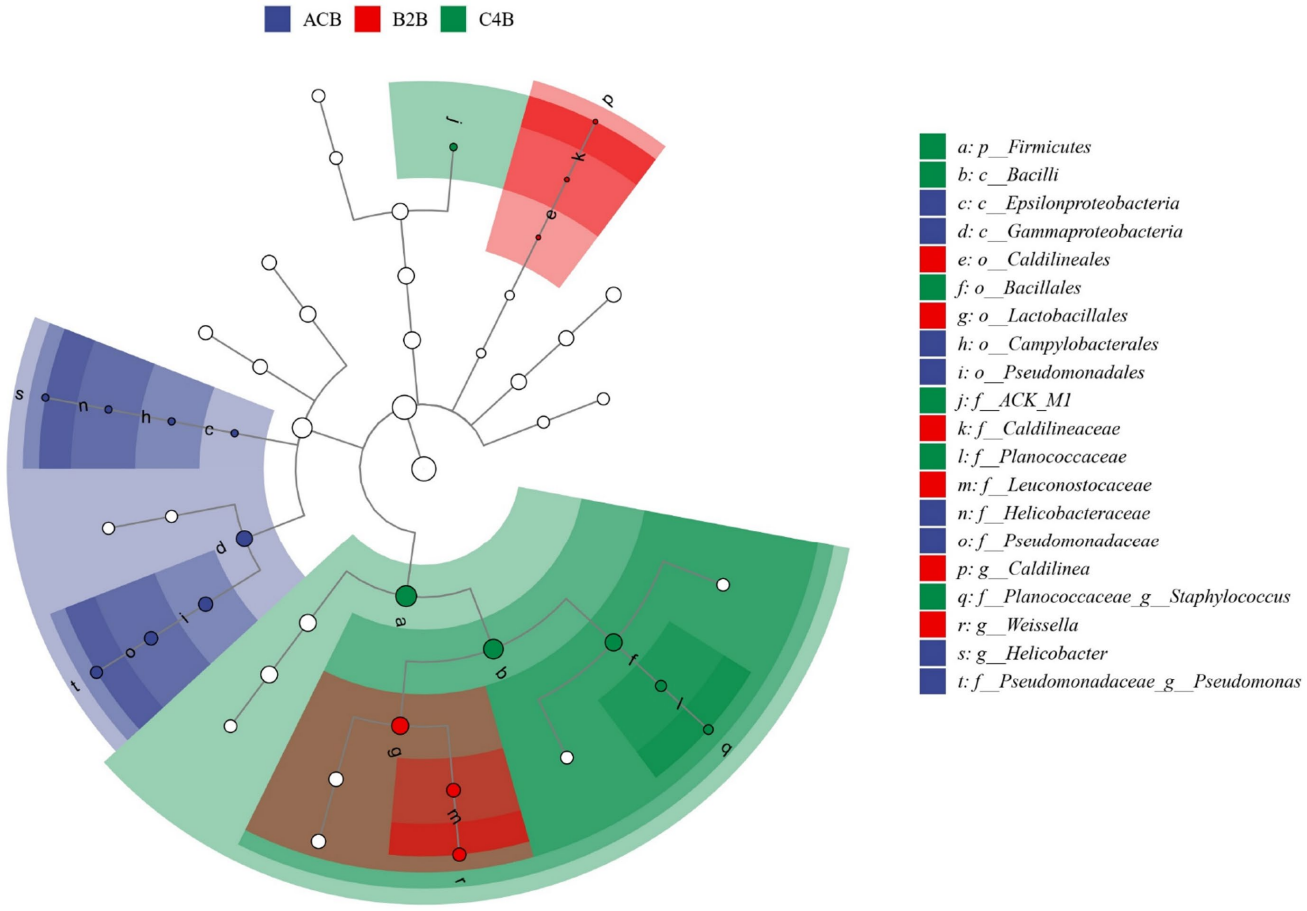
The LEfSe dendrogram represents the hierarchical organization of central taxonomic units from phylum to genus (from inner to outer circles) present within the community samples. The size of the nodes correlates with the average relative abundance of the respective taxonomic unit. Hollow nodes represent taxonomic units with non-significant (*p* value > 0.05) intergroup differences, whereas other colored nodes signify the taxa presenting significant (*p* value <0.05) group disparities. Alphabet labels denote the names of taxonomic units with significant (*p* value <0.05) intergroup variation.

Meanwhile, we employed Linear Discriminant Analysis Effect Size (LEfSe) analysis to discern the critical discriminatory species between different groups robustly, often referred to as our biomarker icon species. Distinct taxa abundance shifts were observed in ACB, B2B and C4B chromium-stressed silkworm gut microbiota. In the control group ACB, *Gammaproteobacteria,* especially *Pseudomonadales*, was more abundant, hinting at their foundational role in an unstressed silkworm gut environment. Conversely, in the chromium-treated groups (B2B and C4B), a decline in *Proteobacteria* abundance was accompanied by a marked rise in *Firmicutes*, notably *Bacilli* of *Weissella* and *Bacillales*. *Weissella paramesenteroides*, a species known to exhibit probiotic properties, showed significance in the B2B group, suggesting an adaptive response (Figure 7). Its presence is an indicator species for chromium stress response, which could offer a mitigation strategy against cytotoxicity induced by metals. In the C4B group, the variety of *Firmicutes* increased to include *Staphylococcus saprophyticus*. Such bacteria, well-suited to endure high osmotic stress and pH extremities, underscores the intensified stress environment in C4B. Moreover, *Actinobacteria* (ACK_M1), known for their role in recalcitrant compound breakdown in C4B, might reflect an attempt to detoxify chromium compounds.

Then, we sought to delve deeply into the range of biological impacts. Based on the functional annotation table retrieved through Picrust2 analysis, we applied a Wilcoxon rank-sum test to draw out variations in the stimulation of diverse KEGG biochemical routes within these three groups (Supplementary Files S5, S6). Among the key findings, the impact on microbiota functionality, particularly on biosynthetic and degradation activities, demonstrated significant changes (*p* value <0.01) in response to chromium stress. The B2B group correlated with a broad suppression of most biosynthetic pathways, particularly those responsible for carbohydrates, cell structure, cofactors, prosthetic groups, electron carriers, and vitamins. Such shifts suggest a scenario of biological unrest, marked by increasing dependence on exogenous resources and dwindling endogenous biosynthetic capacities.

Conversely, the C4B group, which endured the maximum chromium concentration, showed a relative boost in all degradation categories, barring aromatic compound degradation. This trend potentially mirrors a stress-triggered metabolic upshift to ensure survival. Chromium exposure fundamentally reshaped fermentation, TCA cycle, and electron transfer processes to counter chromium-induced stress. Chromium impedes normal metabolic activities, with more significant concentrations amplifying these disturbances. A notable rise in antibiotic resistance represented a defensive countermeasure, suggesting an innate protective strategy to maintain microbiota equilibrium amidst the chromium-triggered environmental upheaval. These alterations have substantial implications for the ecological balance of gut microbiota and, consequently, the physiological performance, stress resilience, and potential biological toxicity of *B. mori*.

**Figure 8:**
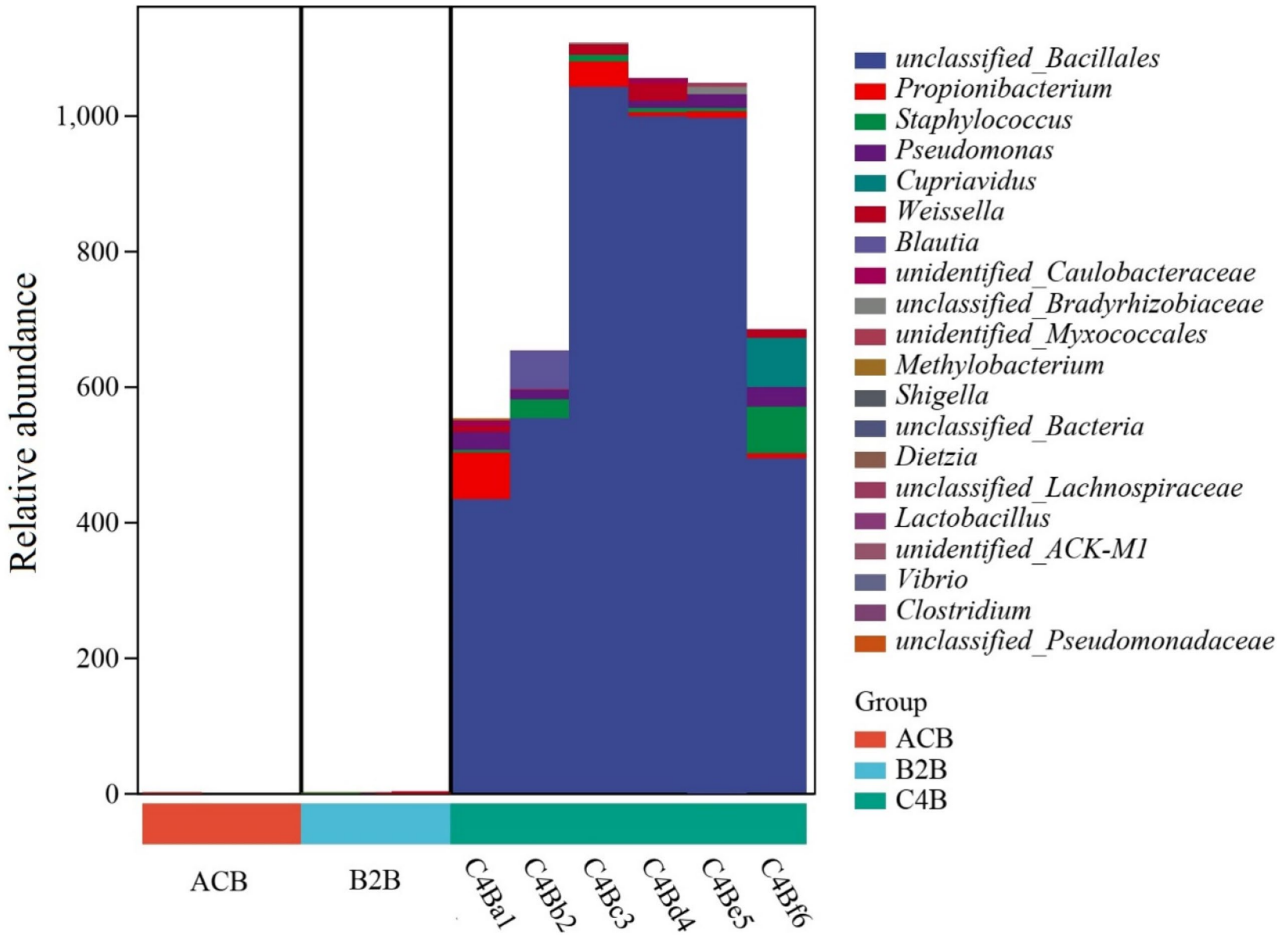
Statistical composition of species contributions to the allantoin degradation IV (anaerobic) pathway. The *x*-axis represents the sample labels, identified by different colors according to their group membership; the *y*-axis displays the relative abundance of the associated allantoin degradation IV (anaerobic) pathway. Contributions to the pathway from various taxonomic units at the taxa level are visualized as layered stacks in varying color gradients.

However, although our study identified several metabolic pathways from various samples, owing to limited sample size and the presence of outliers, no meaningful differences were observed in most of these pathways. We conducted species composition analysis of KEGG pathways using a stratified sample metabolic pathway abundance table. Among these, only the allantoin degradation IV (anaerobic) pathway was significantly upregulated (*p* < 0.05) in the C4B group as compared to both B2B and ACB groups. The observed upregulation of the allantoin degradation IV pathway in the C4B group sheds light on the notable metabolic changes this group is undergoing. This group potentially resorts to anaerobic allantoin degradation due to changing microenvironmental conditions. Allantoin degradation, primarily known for its role in nitrogen metabolism under anaerobic conditions, may indicate an alternative metabolic adaptation of the organisms within the C4B group. While we determined the composition of this pathway, a substantial amplification of the unclassified_*Bacillales* was detected (Figure 8). This observation could suggest that these bacteria have developed a survival strategy in conditions marked by increasing chromium concentrations, as inferred from their scarceness or complete absence within groups ACB and B2B. Similarly, the noticeable propagation of *Weissella* within both B2B and C4B cohorts was found repetitively, which once again denotes a probable role for this bacterium in responding to chromium-induced perturbations. Additionally, some bacterial strains, such as *Cupriavidus* and unidentified_*Myxococcales,* appear to be exclusive residents of the C4B group, potentially indicating their detoxification functionality within high-chromium environments.

### Non-targeted metabolomics characterization and comparative analysis

**Figure 9:**
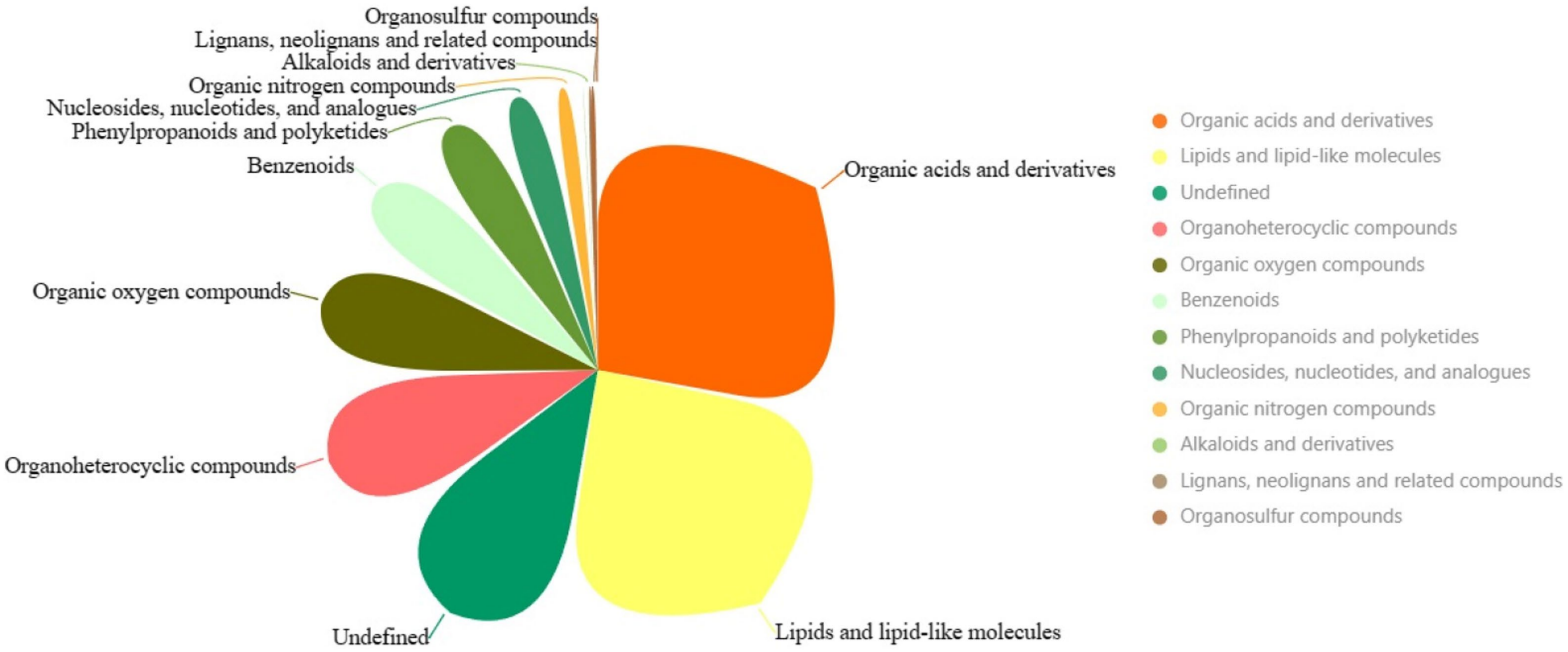
The pie chart illustrates the superclasses of metabolites. Larger areas represent more dominant categories.

Another pivotal cornerstone supporting our study is underpinned by comprehensive metabolomics analysis, illuminating the interplay between the host and its microbiome and delineating the contribution of these interactions to the studied physiological phenomena. The overall metabolomic profile highlights various superclasses of metabolites identified in the intestinal microbiota following exposure to chromium. The three most dominant metabolite superclasses are “Organic acids and derivatives” (634 metabolites), “Lipids and lipid-like molecules” (546 metabolites), and an undefined category (277 metabolites) (Figure 9; Supplementary File S7). The copious diversity and quantities of “Organic acids and derivatives” suggest a high metabolic turnover involving carboxylic acids, esters and carbonyl groups, potentially related to energy production, detoxification, and chromium resistance. “Lipids and lipid-like molecules” superclass, being second abundant, might imply modifications of structural constituents of cell membranes and signaling molecules, which could be instrumental in perpetuating the chemical defences against chromium-induced stress. Perhaps signaling cascades initiated under chromium exposure might be regulated by lipid-mediated pathways, an area worth exploring. The least represented categories, such as “Lignans, neolignans and related compounds” and “Organosulfur compounds,” might hint at their less significant roles in managing chromium-related disturbances. However, their relatively low abundance must not be mistaken as lacking biological significance, considering they might possess specialized functions rather than broad-spectrum activity.

Then, our study delineates the wide-ranging ramifications on the metabolome, examined across three experimental tiers. We undertook a comparative analysis of distinctions in Variable Importance in the Projection (VIP) and fold change (FC) within the ACB, B2B, and C4B clusters about metabolite fluctuations. The VIP score delineates the significance of a metabolite in discriminating between groupings, while fold change illuminates the extent of alteration in metabolite concentrations between two groups. Noteworthy divergences in VIP scores and fold changes associated with specific metabolites could elucidate chromium’s biological implications on the silkworms’ gut metabolism, encapsulating functional, physiological, and ecological facets, stress resistance, and physical toxicity. The ACB collective served as a reference, whereas the B2B and C4B factions represent moderate and high chromium exposure, respectively (Supplementary File S8).

A collection of metabolites demonstrated elevated VIP scores and fold changes within the B2B cohort in contrast to the ACB group, including D-mannitol 1-phosphate, L-iditol, glutamine, Glutaraldehyde, asparagine, urocanic acid, glutamic acid, Histidine, dihydropyrimidine, and L-gulono-1,4-lactone. These disparities in metabolite concentrations intimate that chromium exposure governs the metabolism of carbohydrates, amino acids, and organic acids in the silkmoth gut. The implicated metabolites partake in many physiological undertakings, such as energy metabolism, antioxidant defence, protein biosynthesis, and signal transduction. Amplified levels of Histidine and dihydropyrimidine might enlighten the mobilization of stress response mechanisms within the B2B configuration (Supplementary File S8).

Upon juxtaposition of C4B and ACB, several metabolites showcased remarkable adjustments in VIP scores and fold changes, including phenol, urocanic acid, D-galactarate, N-fructosyl pyroglutamate, 17,20-dimethylprostaglandin F1.alpha., and gentisic acid. These perturbations emphasize the influence of intensified chromium exposure on gut metabolism. Phenol, urocanic acid, and D-galactarate are linked with oxidative stress and inflammation; N-fructosyl pyroglutamate and 17,20-dimethyl prostaglandin F1.alpha. Possibly contributes to regulating gut barrier function and immune response, and gentisic acid is associated with detoxification routes and antioxidant processes. A comparison between B2B and C4B cohorts unraveled additional metabolites with significant shifts (*p* < 0.01) in VIP scores and fold changes, encompassing a range of amino acids, organic acids, and carbohydrates. These metabolites comprise glutamine, dihydrouracil, 4-ketopimelic acid, Lpc 18:2, Histidine, and L-pipecolic acid. Glutamine, dihydrouracil, and L-pipecolic acid correlate with stress response, while 4-keto pimelic acid and Lpc 18:2 might engage in lipid metabolism. Histidine is crucial in antioxidant defence, immune modulation, and protein synthesis. The differential metabolite profiles discerned from this study indicate that chromium exposure interferes with diverse aspects of silkworm gut metabolism. The identified metabolites participate in various biological functions, incorporating energy metabolism, stress response, immune regulation, and antioxidant events. Fluctuations in metabolite concentrations responsive to chromium exposure suggest potential adaptations within the silkworms’ gut to chromium-induced stress (Supplementary File S9).

**Figure 10:**
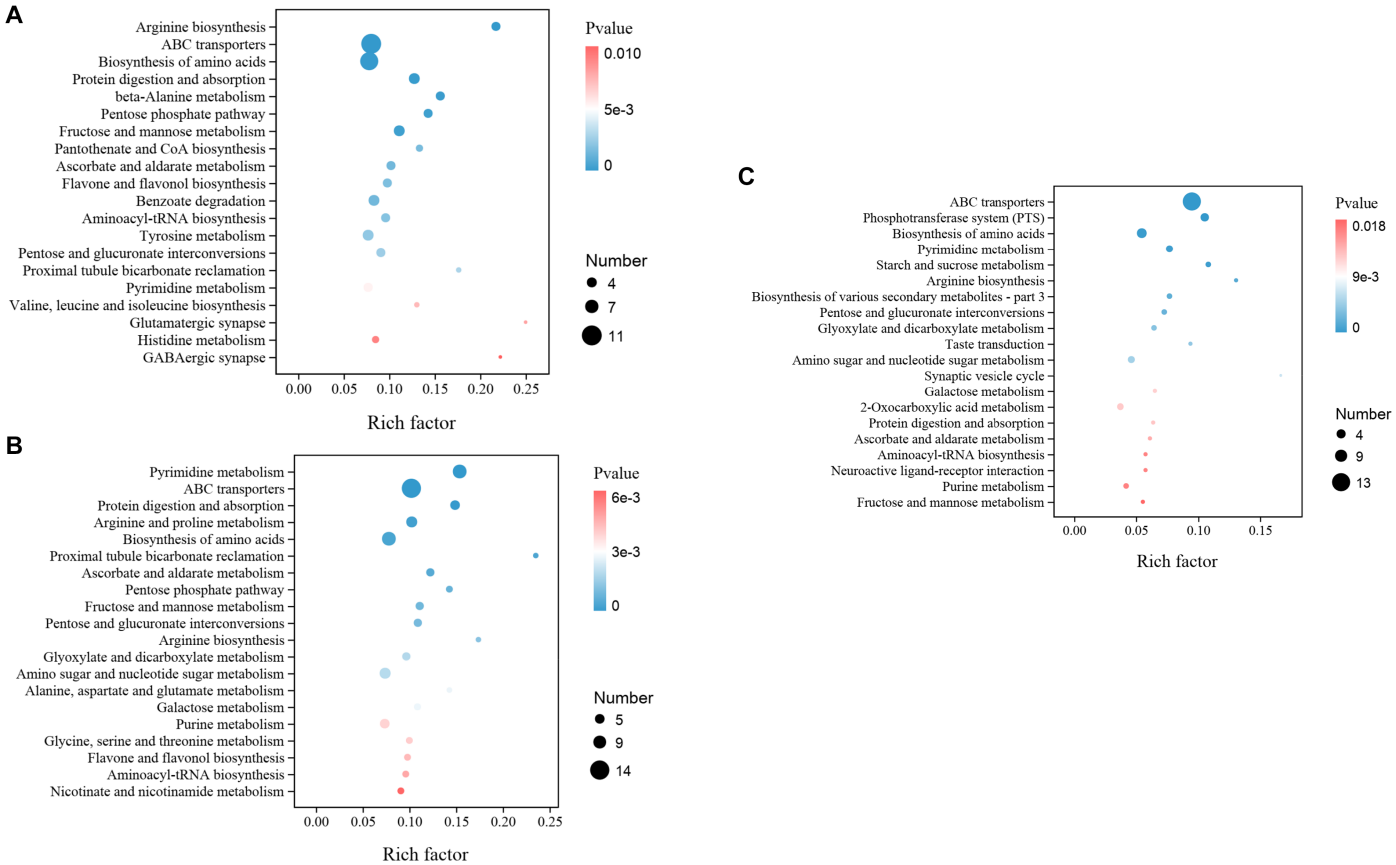
The KEGG enrichment map illustrated the top different metabolic functions among groups. **(A)** B2B vs. ACB; **(B)** C4B vs. ACB; **(C)** C4B vs. B2B. The number of genes and *p*-values are shown.

Moreover, while we assign the KEGG functional annotations to these differential metabolites, the same pattern with the gut microbiota is observed where B2B and C4B groups manifest significant deviations in an array of metabolic trajectories when contrasted against ACB, accentuating the biological relevance of metabolite inconsistencies. A prominent observation is stress-induced dysregulation. Essential pathways instrumental for amino acid biosynthesis, particularly the ‘Arginine biosynthesis’ and ‘Valine, leucine, and isoleucine biosynthesis’, underwent substantial perturbations, indicative of a pivotal regulation and redistribution of amino acid metabolism in response to chromium. Analogously, ‘ABC transporters’, ‘Protein digestion and absorption’, and ‘Biosynthesis of amino acids were significantly enriched (*p* value <0.05), signifying a potential physiological adaptation aimed at attenuating the biomechanical hazards (Figure 10A). Moreover, the metabolic machinery regulating sugar metabolism displayed tangible transformation, signaled by alterations in pathways like the ‘Pentose Phosphate Pathway’, ‘Fructose and Mannose Metabolism’, and ‘Pentose and glucuronate interconversions, providing evidence of metabolic reconfiguration for energy homeostasis under duress. A marked upregulation in detoxifying and oxidative stress-responsive networks, such as ‘Ascorbate and aldarate metabolism’ and ‘Flavone and Flavonol biosynthesis’, paints a vivid portrayal of the orchestrated defensive measures (Figure 10B). Continuing our comparative analysis between C4B and B2B, an escalating trend in these metabolic distortions is apparent as chromium dosage surges, with newer, hitherto insignificant pathways like the ‘Phosphotransferase system (PTS)’, ‘Glyoxylate and dicarboxylate metabolism’, and ‘Neuroactive ligand-receptor interaction’ claiming prominence (Figure 10C; Supplementary File S10).

Subsequently, after dual validation of our metabolomics data against microbial abundance through PCA and CIA analyses (Supplementary Figures S1, S2), we computed the Spearman rank correlation coefficients between these two sets. The correlation matrix results initially show a pervasive association between the two types of omics data, projecting a multiplex relationship between bacterial species and metabolic products, with numerous significant correlations (*p* < 0.05) present (Supplementary File S11). With a meticulous inspection, we identified our previously pointed out marker of differential bacterial species, such as *Weissella*, *Thermus*, *Geobacillus*, etc., as they had substantial connections with various types of differential metabolic products. These species are considered to have a crucial functional impact.

**Figure 11:**
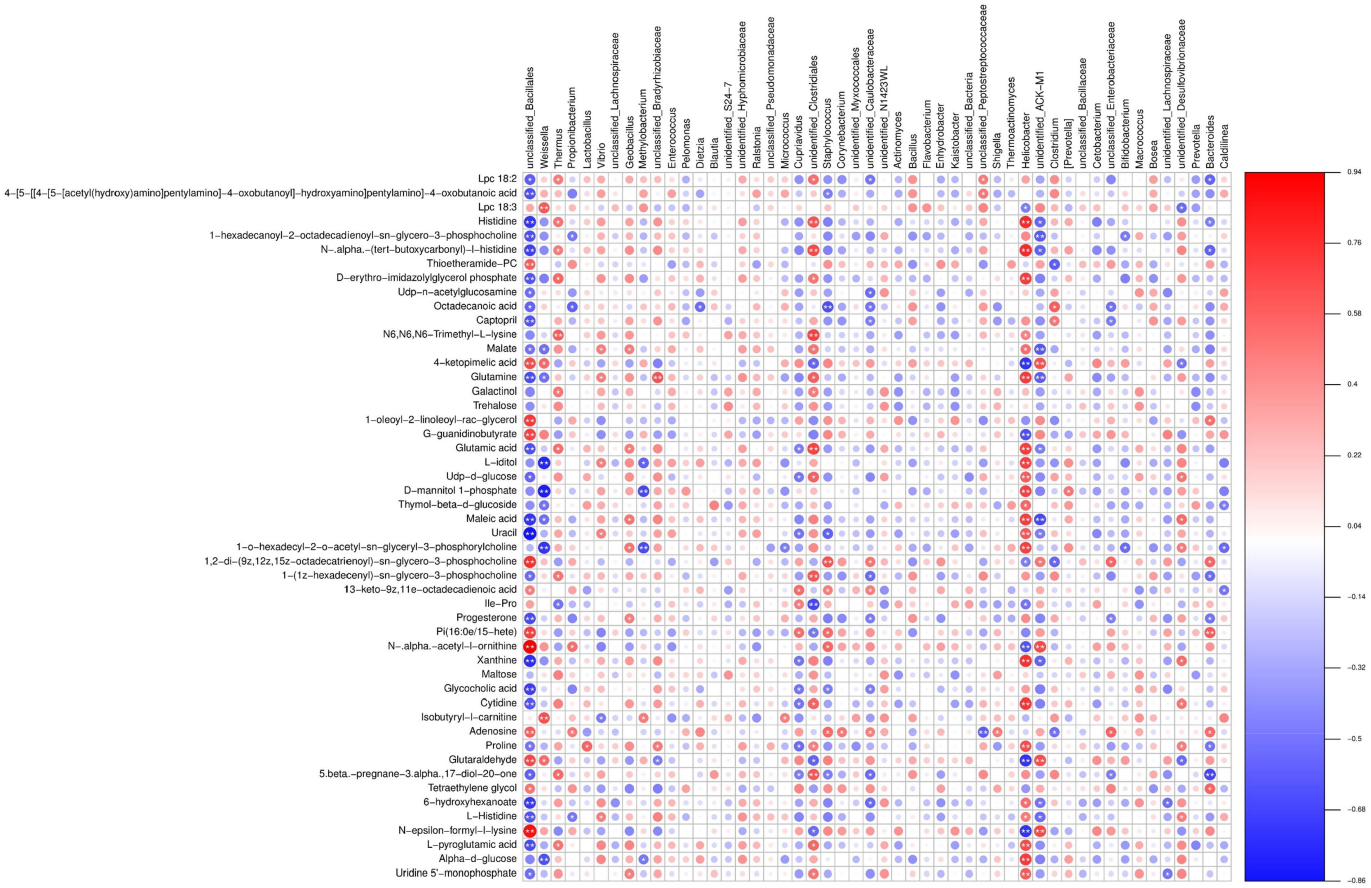
Heatmap illustrating the correlation between microbial species and metabolomic profiles. A positive correlation is depicted in red, while a negative correlation is shown in blue. The color intensity represents the strength of correlation. The pairs of “microbiota–metabolite” demonstrating significant association (*p* < 0.05) are indicated by asterisks.

A comprehensive analysis of the presented data reveals a complex interplay between different bacterial species and metabolites, shedding light on its biological significance in the silkworm gut microbiota under chromium stress. Notably, *Weissella* and *Pelomonas* represented the marker species with distinct patterns of association with various metabolites. *Weissella* exhibited a positive correlation with metabolites such as Glutaraldehyde (0.5418), 4-keto pimelic acid (0.5501), and N-epsilon-formyl-l-lysine (0.3209), while negatively correlating with N-.alpha.-(tert-butoxycarbonyl)-l-histidine (−0.4262) and Histidine (−0.3870) (Figure 11; Supplementary File S11). These patterns suggest a potential role of *Weissella* in enzyme regulation and synaptic processes that are critical under chromium exposure. Conversely, *Pelomonas*, though demonstrating relatively weaker correlations, showed a possible involvement in lipid metabolism, such as its positive relationship with Isobutyryl-l-carnitine (0.0113) and a negative one with Lpc 18:2 (−0.2910). The function of *Pelomonas* in lipid metabolism may underscore its potential in maintaining homeostasis and chromium toxicity resistance. Beneficial bacteria such as *Weissella*, *Propionibacterium*, *Methylobacterium*, *Cupriavidus*, and *Bifidobacterium* displayed notably strong correlations with metabolites like Glutaraldehyde, 4-ketopimelic acid, N-epsilon-formyl-l-lysine, and N-.alpha.-(tert-butoxycarbonyl)-l-histidine. These correlations suggest that these metabolites might modulate gut microbiota under chromium stress. Notably, *Weissella* showed a strong association with Isobutyryl-l-carnitine and Lpc 18:3 metabolites. *Propionibacterium* had a tightly coupled relationship with N-epsilon-formyl-l-lysine and 1-oleoyl-2-linoleoyl-rac-glycerol. *Methylobacterium* exhibited notable correlations with Glutaraldehyde and 4-ketopimelic acid. *Cupriavidus* was highly associated with Glutaraldehyde, 4-ketopimelic acid, and N-. Alpha. -acetyl-l-ornithine metabolites and *Bifidobacterium* revealed connections with N-epsilon-formyl-l-lysine and Isobutyryl-l-carnitine. The potential implications of these associations are speculated to indicate the biological function of chromium within the silkworm gut environment (Figure 11). The association of aromatic metabolites such as Glutaraldehyde with *Methylobacterium* may suggest the biotransformation capability and metabolic versatility of this bacterium upon chromium exposure. Likewise, the changes associated with isobutyryl carnitine and *Weissella* might underscore an elevated stress response and potential toxicity. Additionally, the dialog between *Cupriavidus* and Glutaraldehyde, or *Bifidobacterium* with acetyl-l-ornithine, might profoundly influence gut homeostasis, proving beneficial in chromium resistance.

## Discussion

Gut microbiota plays a crucial role in influencing the health and wellbeing of the host organism (Ceja-Navarro et al., 2015; Aksoy, 2018; Wang et al., 2020). In this study, we investigated the changes in gut microbiota composition in response to varying levels of chromium stress in *B. mori*. Our findings provide valuable insights into the mechanisms underlying gut microbiota structure in different groups and shed light on the potential adaptations of gut microbiota to chromium stress.

We analyzed the abundance chart of the microbiome to identify substantial variations in microbial diversity across different experimental groups. The results highlighted the integral role of chromium in shaping the bacterial community within the gut of silkworms. The control group (ACB) exhibited dominance of specific microbial taxa, such as *Thermus*, *Geobacillus*, and *Bacillus*, which are likely involved in maintaining the normal physiological state of silkworms under unstressed conditions. In the groups subjected to chromium stress (B2B and C4B), we observed alterations in the prevalence of specific taxa. The abundance of *Weissella* increased in the B2B group, while the prevalence of *Thermus* decreased, suggesting a potential chromium resistance or detoxification capability in *Weissella*. Similarly, in the C4B group with high chromium exposure, *Propionibacterium* showed a marked increase, while *Weissella* decreased. These shifts in microbial populations indicate potential adaptive modifications to cope with varying levels of chromium stress (Cava et al., 2009; Teixeira et al., 2021; Ahmed et al., 2022).

We employed Linear Discriminant Analysis Effect Size (LEfSe) analysis further to dissect the key discriminatory species between different groups. This analysis revealed distinct shifts in the abundance of taxa among the ACB, B2B, and C4B groups. In the control group ACB, we observed a higher quantity of *Gammaproteobacteria*, particularly *Pseudomonadales*, which may contribute to the healthy gut profile of silkworms in the absence of stress (Keller-Costa et al., 2014; Lin et al., 2016). In contrast, the chromium-treated groups (B2B and C4B) showed a decline in Proteobacteria abundance alongside an increase in Firmicutes, specifically *Weissella* (Fusco et al., 2015; Teixeira et al., 2021) and *Bacillales* (Santana et al., 2016; Daquila et al., 2021). *Weissella paramesenteroides*, a species known for its probiotic properties (Libonatti et al., 2019; Prado et al., 2020), displayed significance in the B2B group, suggesting an adaptive response to chromium stress. In the C4B group, the abundance of Firmicutes expanded to include *Staphylococcus saprophyticus* (Golledge, 1988; Ehlers and Merrill, 2023), indicating a more intense stress environment. Actinobacteria (ACK_M1) in the C4B group suggests an attempt to detoxify chromium compounds. These findings highlight the importance of specific microbial taxa in responding to chromium stress and potentially contributing to the protection and adaptation of the host organism.

Furthermore, we investigated the functional impact of chromium stress on gut microbiota by analyzing the stimulation of diverse KEGG biochemical pathways using Picrust2 analysis. The results revealed substantial changes in biosynthetic and degradation activities in response to chromium stress, consistent with previous acknowledgements (Vincent and Lukaski, 2018; Vincent, 2019; Li Y. et al., 2022). The B2B group showed suppression of most biosynthetic pathways, indicating an increased reliance on external resources and reduced endogenous biosynthetic capacities. On the other hand, the C4B group, exposed to high chromium concentrations, exhibited a relative boost in degradation pathways, excluding aromatic compound degradation. This shift suggests a metabolic upshift to ensure survival under stressful conditions. Chromium exposure also reshaped fermentation, TCA cycle, and electron transfer processes, likely as a response to counteract chromium-induced stress. Notably, an increase in antibiotic resistance was observed, potentially serving as a defensive countermeasure to maintain microbiota equilibrium in the presence of chromium stress. These alterations have significant implications for the ecological balance of gut microbiota and the physiological performance, stress resilience, and potential biological toxicity of silkworms.

Although we identified several metabolic pathways, limitations such as small sample size and the presence of outliers prevented crucial differences in most of these pathways. However, the allantoin degradation IV (anaerobic) pathway was notably upregulated in the C4B group compared to the B2B and ACB groups, indicating metabolic adaptations to changing microenvironmental conditions. The amplification of *unclassified_Bacillales* in the C4B group suggests a survival strategy in states with increasing chromium concentrations. Comprehending the relationship between allantoin degradation and *Bacillales*, an order of Gram-positive bacteria offers fresh insights into their metabolic versatility and resilience in hostile environments (Martínez-Gómez et al., 2014). The latest investigations have shown that several *Bacillales* members harbor exceptional enzymatic machinery enabling them to degrade allantoin, a nitrogen-rich compound produced from purine metabolism (Martínez-Gómez et al., 2014; Ma et al., 2016; Li et al., 2017). One pivotal gene locus, the UreD cluster, reportedly catalyzes allantoin degradation. Using *Bacillus subtilis* as a representative of *Bacillales*, researchers have empirically identified the presence of this gene cluster. Its possession confers an adaptive advantage to the bacterium, particularly in nitrogen-deficient settings, enabling allantoin to serve as an alternative nitrogen source (Li et al., 2017). A dedicated biological examination of *Bacillus subtilis* implicates that this bacterium selectively metabolizes allantoin during nutrient deprivation, as manifested by the amplified expression of associated enzymes, such as Allantoicase and Uricase (Ma et al., 2016). The fundamental mechanism underscoring this is the detoxification process, which sees the decomposition of allantoin into simpler, non-toxic compounds, thereby minimizing potential oxidative stress that can harm bacterial cells. Through such allantoin detoxifying pathways, *Bacillales* present metabolic flexibility, capable of withstanding nitrogen scarcity by switching their nitrogen source. This breakthrough understanding unravels a part of the survival strategy of bacteria in hostile conditions, proving potentially beneficial in understanding the role of microorganisms in biogeochemical nitrogen cycles. The repeated propagation of *Weissella* in both B2B and C4B groups suggests its potential role in response to chromium-induced perturbations. Additionally, specific bacterial strains such as *Cupriavidus* and *unidentified_Myxococcales* (Schäberle et al., 2014; Cao et al., 2019) were exclusively found in the C4B group, potentially indicating their detoxification functionality in high-chromium environments.

In response to the escalating problem of non-biodegradable heavy-metal contamination, bioremediation via biological agents, particularly *Bacillales*, has emerged as a potentially effective alternative to traditional physicochemical decontamination methods (Wróbel et al., 2023). Studies reveal that *Bacillales* bear exceptional heavy-metal detoxifying properties, enabling their practical use in remediating contaminated environments. Research demonstrates *Bacillales*’ ability to facilitate heavy-metal ion adsorption by forming biofilms and biosorption mechanisms—experiences with *Bacillus* sp. *GH-s29* and *Bacillus* spp. *CPB4* strain provides proof of concept, each successfully removing heavy-metal ions from water systems (Maity et al., 2023). The heavy-metal resistance genes present in species like *Bacillus oceanisediminis* 2,691 underline the genetic basis for this detoxification (Kim et al., 2007; Maity et al., 2023). Moreover, *Bacillus cereus RC-1* has been shown to bioaccumulate heavy metals while releasing cations, potentially benefiting plant growth in contaminated soils. This phenomenon was observed in an experiment with Pakchoi plants, which showed increased biomass and reduced Cd and Pb uptake when grown with heavy metal-immobilizing bacteria (Jung et al., 2016; Huang F. et al., 2018; Han et al., 2020). These findings highlight *Bacillales*’ significant potential for developing bioremediation solutions for heavy-metal contamination. However, current studies are predominantly laboratory-scale, necessitating field-based, long-term studies on their practical efficacy. Further research into specific detoxifying mechanisms of *Bacillales* to enhance their functions, coupled with developing technologies for their selective growth and application, could significantly elevate our ability to manage heavy-metal contamination and mitigate associated risks.

Nevertheless, we employed non-targeted metabolomic characterization to cope with the microbiota analyses, devising an insightful perspective on the metabolic landscape amidst varied chromium exposure. The metabolites portraying differential concentrations paint a compelling picture of energy metabolism, stress response, immune regulation, and antioxidant processes, embodying the multifaceted nature of their response. Prominently, metabolites such as D-mannitol 1-phosphate, L-iditol and glutamine demonstrated by VIP scores and fold changes were under the B2B group. These metabolites hold a pivotal role in carbohydrate and amino acid metabolism, antioxidant defence, and protein biosynthesis (Mancera et al., 2002; Oosaka, 2009; Cruzat et al., 2018; Coqueiro et al., 2019; Nguyen et al., 2019), thus attesting to the metabolic flexibility of silkworms. Under the C4B group, we observed significant shifts in metabolite concentrations, underscoring increased oxidative stress, inflammation, and an enhanced detoxification and antioxidant response. Moreover, we noticed a direct correlation between the increased chromium dosage and the degree of metabolic distortions, inferring adaptive changes in the metabolic landscape. Importantly, functional annotation via the KEGG highlighted the considerable impact on metabolic pathways, mirroring the transformations ignited by chromium stress.

Exploring the association of bacterial abundance and metabolic alteration, we identified an intricate network underlining the interrelation between diverse bacterial species and their metabolic byproducts, emphasizing their functional significance during chromium exposure. Certain bacterial species, namely *Weissella*, *Pelomonas*, *Propionibacterium*, *Methylobacterium*, *Cupriavidus* and *Bifidobacterium*, exhibited strong correlations with differential metabolites, hinting at their potential contribution in chromium detoxification, enzyme regulation, lipid metabolism, antioxidants production, stress response and homeostasis maintenance (Cava et al., 2009; Keller-Costa et al., 2014; Lin et al., 2016; Dong et al., 2019; Prado et al., 2020; Daquila et al., 2021; Wurm et al., 2023).

In conclusion, our study constitutes a pioneering attempt to decipher gut microbiota-metabolite interactions under chromium stress, shaping the blueprint for an integrated understanding of heavy metal-induced ecological disruption. Our findings offer new insights into the dynamic and evolving association between the gut microbiome and metabolic changes under chromium stress in silkworms.

## Data availability statement

The data presented in the study are deposited in the GSA (Genome Sequence Archive) repository, accession number PRJCA019204.

## Ethics statement

Ethical approval was not required for the study involving animals in accordance with the local legislation and institutional requirements because silkworms is a common insect, and it is also a model species in scientific research.

## Author contributions

Y-ZC: Data curation, Writing – original draft. W-TR: Investigation, Validation, Writing – review & editing. Y-CQ: Data curation, Investigation, Writing – original draft. L-YL: Data curation, Investigation, Writing – original draft. JL: Data curation, Investigation, Writing – original draft. M-JL: Data curation, Investigation, Writing – original draft. LX: Formal analysis, Investigation, Resources, Writing – original draft. X-DL: Writing – original draft, Writing – review & editing, Funding acquisition, Project administration, Supervision. D-LG: Conceptualization, Writing – original draft, Writing – review & editing.

## Footnotes

^1^ https://www.genescloud.cn/
