# Supplementary figures and images for: Integrative analysis of microbiota and metabolomics in chromium-exposed silkworm (Bombyx mori) midguts based on 16S rDNA sequencing and LC/MS metabolomics
Source: Front Microbiol. 2023 Oct 25;14:1278271. doi: 10.3389/fmicb.2023.1278271 (PMC10635416; doi:10.3389/fmicb.2023.1278271)

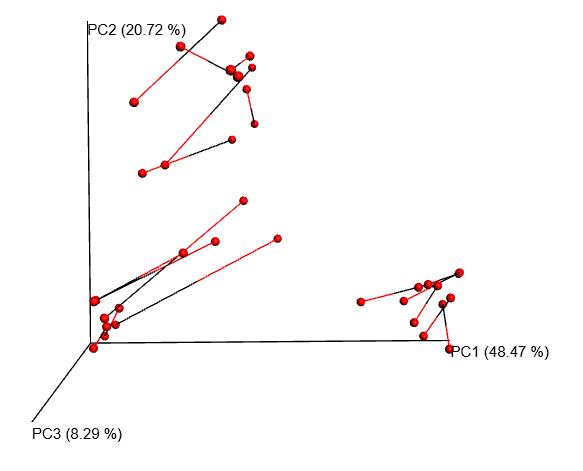

Supplement: Supplementary file 12 [file Image_1.JPEG]

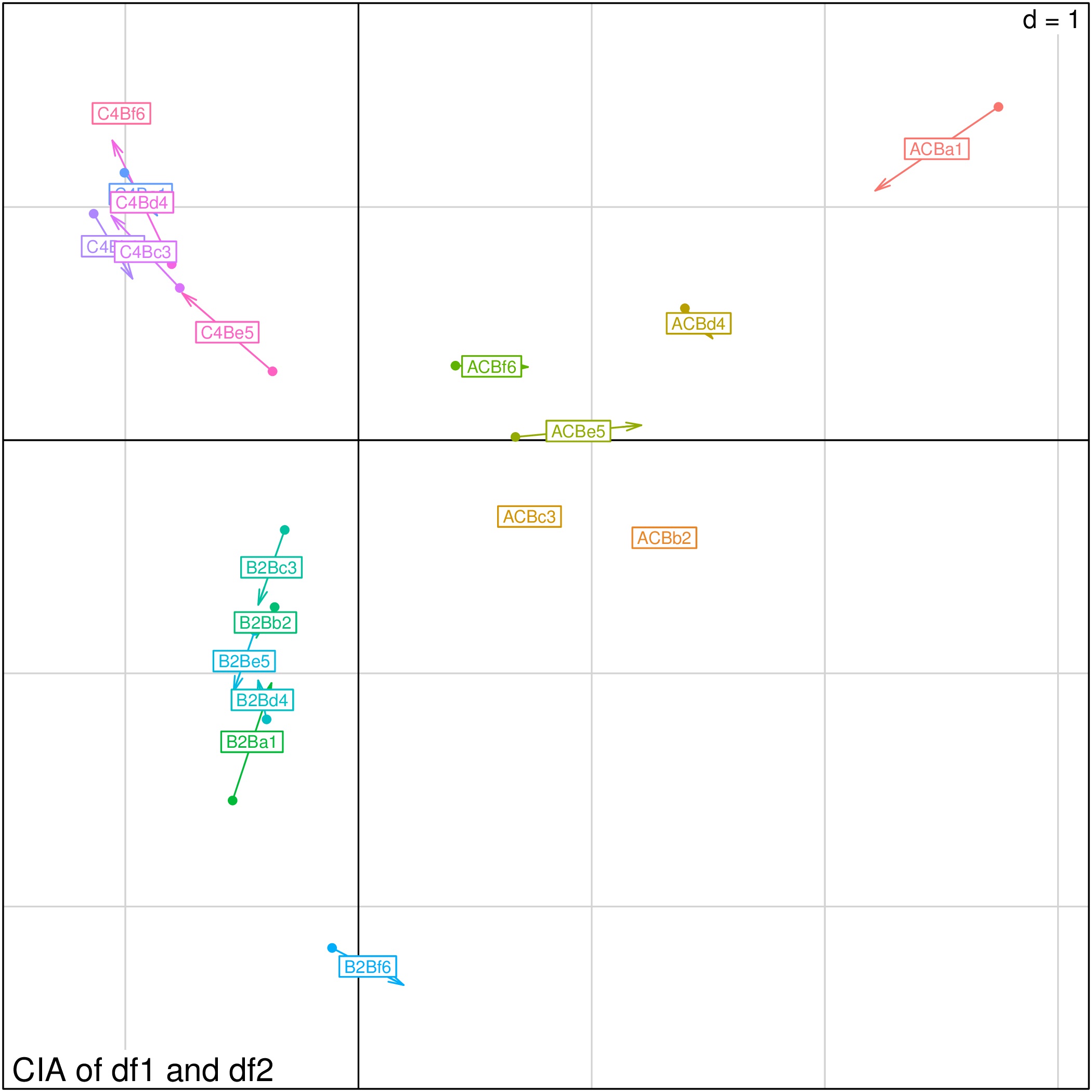

Supplement: Supplementary file 13 [file Image_2.JPEG]
